# Immediate Effects of Smoking on Cardiorespiratory Responses During Dynamic Exercise: Arm Vs. Leg Ergometry

**DOI:** 10.3389/fphys.2015.00376

**Published:** 2015-12-10

**Authors:** Chien-Liang Chen, Jing-Shia Tang, Ping-Chia Li, Pi-Ling Chou

**Affiliations:** ^1^Department of Physical Therapy, I-Shou UniversityKaohsiung, Taiwan; ^2^Department of Nursing, Chung-Hwa University of Medical TechnologyTainan, Taiwan; ^3^Institute of Allied Health Sciences, National Cheng Kung UniversityTainan, Taiwan; ^4^Department of Occupational Therapy, I-Shou UniversityKaohsiung, Taiwan; ^5^School of Nursing, Kaohsiung Medical UniversityKaohsiung, Taiwan

**Keywords:** acute effect, cigarette smoke, aerobic exercise, cardiopulmonary exercise test, cycling ergometry, heart rate variability, pulmonary function test

## Abstract

**Purpose:** This study compared the immediate effects of smoking on cardiorespiratory responses to dynamic arm and leg exercises.

**Methods:**This randomized crossover study recruited 14 college students. Each participant underwent two sets of arm-cranking (AC) and leg-cycling (LC) exercise tests. The testing sequences of the control trial (participants refrained from smoking for 8 h before testing) and the experimental trial (participants smoked two cigarettes immediately before testing) were randomly chosen. We observed immediate changes in pulmonary function and heart rate variability after smoking and before the exercise test. The participants then underwent graded exercise tests of their arms and legs until reaching exhaustion. We compared the peak work achieved and time to exhaustion during the exercise tests with various cardiorespiratory indices [i.e., heart rate, oxygen consumption (VO_2_), minute ventilation (V_E_)]. The differences between the smoking and control trials were calculated using paired *t*-tests. For the exercise test periods, VO_2_, heart rate, and V_E_ values were calculated at every 10% increment of the maximal effort time. The main effects of the time and trial, as well as their trial-by-time (4 × 10) interaction effects on the outcome measures, were investigated using repeated measure ANOVA with trend analysis.

**Results:** 5 min after smoking, the participants exhibited reduced forced vital capacities and forced expiratory volumes in the first second (*P* < 0.05), in addition to elevated resting heart rates (*P* < 0.001). The high-frequency, low-frequency, and the total power of the heart rate variability were also reduced (*P* < 0.05) at rest. For the exercise test periods, smoking reduced the time to exhaustion (*P* = 0.005) and the ventilatory threshold (*P* < 0.05) in the LC tests, whereas no significant effects were observed in the AC tests. A trend analysis revealed a significant trial-by-time interaction effect for heart rate, VO_2_, and V_E_ during the graded exercise test (all *P* < 0.001). Lower VO_2_ and V_E_ levels were exhibited in the exercise response of the smoking trial than in those of the control LC trials, whereas no discernable inter-trial difference was observed in the AC trials. Moreover, the differences in heart rate and V_E_ response between the LC and AC exercises were significantly smaller after the participants smoked.

**Conclusion:** This study verified that smoking significantly decreased performance and cardiorespiratory responses to leg exercises. However, the negative effects of smoking on arm exercise performance were not as pronounced.

## Introduction

The immediate detrimental effects of cigarette smoking on pulmonary and cardiovascular function, particularly when these systems are stressed by the metabolic demands of exercise, have been thoroughly investigated. The inhalation of cigarette smoke exerts the immediate respiratory effect of increasing airway resistance (Sobol et al., 1977; Iyawe et al., 2007). Moreover, carbon monoxide levels in the blood increase after smoking (Seppänen, 1977; Hawari et al., 2013). Both of these changes reduce the amount of oxygen absorbed into the body. Several studies have shown that cigarette smoking significantly lowers exercise capacity, maximal oxygen consumption (VO_2max_), and the anaerobic threshold. In addition, smoking's immediate effects have been found to increase the heart rate at rest and to decrease the heart rate during maximal exercise (Klausen et al., 1983; Hirsch et al., 1985; Rotstein and Sagiv, 1986). However, the aforementioned studies have used only lower-body exercises such as treadmill running or leg cycling (LC), and previous studies in general have seldom focused on upper-body exercises. The immediate effects of cigarette smoking on arm-cranking (AC) exercise, for example, have not been reported.

The physiologic work capacities of the upper and lower extremities limit maximal exercise performance differently. At any level of VO_2_, both the heart rate and blood pressure are higher during arm exercise than during leg exercise (Power and Howley, 2004). In addition, other researchers have found that substrate oxidation during arm exercise differs from substrate oxidation during leg exercise. Ahlborg and Jensen-Urstad (1991) reported that dependence on plasma glucose as an energy source is higher during AC exercise than during LC exercise. This difference in substrate oxidation patterns may be due to discrepancies in the distribution of muscle fiber types. Specifically, upper-body musculature has a greater percentage of fast-twitch fibers compared with lower-body musculature (Johnson et al., 1973). Moreover, previous studies have suggested that work performed by the upper body might interfere with the normal recruitment of respiratory muscles. Competing demands for the respiratory muscles may result in respiratory limitations or prevent a person from properly performing upper body exercise (Cerny and Ucer, 2004). The present study was designed to determine the acute effect of smoking on cardiorespiratory responses to dynamic arm and leg exercises. We hypothesized that the negative effects of smoking would differ significantly between these two types of exercise.

## Methods

### Participants

Sixteen moderately trained males initially volunteered to participate in this study. Two participants failed to complete all of the sessions because for personal reasons and were therefore excluded from the final analysis, leaving 14 participants with a mean age, height, weight, and body mass index were 20.4 ± 1.5 years, 171.8 ± 6.1 cm, 68.3 ± 6.2 kg, and 23.2 ± 2.7 kg/m^2^, respectively. The participants had to be regular smokers and involved in organized sports. Their mean duration of smoking was approximately 4.0 ± 2.0 years, and the mean number of cigarettes smoked per day was 9.9 ± 2.9. All participants were routinely involved (6.1 ± 1.8 h/wk) in various intermittent activities (e.g., volleyball, soccer, basketball, or softball) and were familiar with maximal training. Medical histories were obtained through direct interviews, and the exclusion criteria were as follows: history of cardiovascular disease, diabetes, or other metabolic disease known to affect the outcome measures; respiratory disease, including asthma, and orthopedic injury preventing the successful completion of the exercise protocols. After a thorough explanation of the study protocol to the participants, written informed consent was obtained. The experimental procedure was in accordance with the Declaration of Helsinki and was approved by the Institutional Review Board at Chung-Hwa University of Medical Technology.

### Design

**Figure 1:**
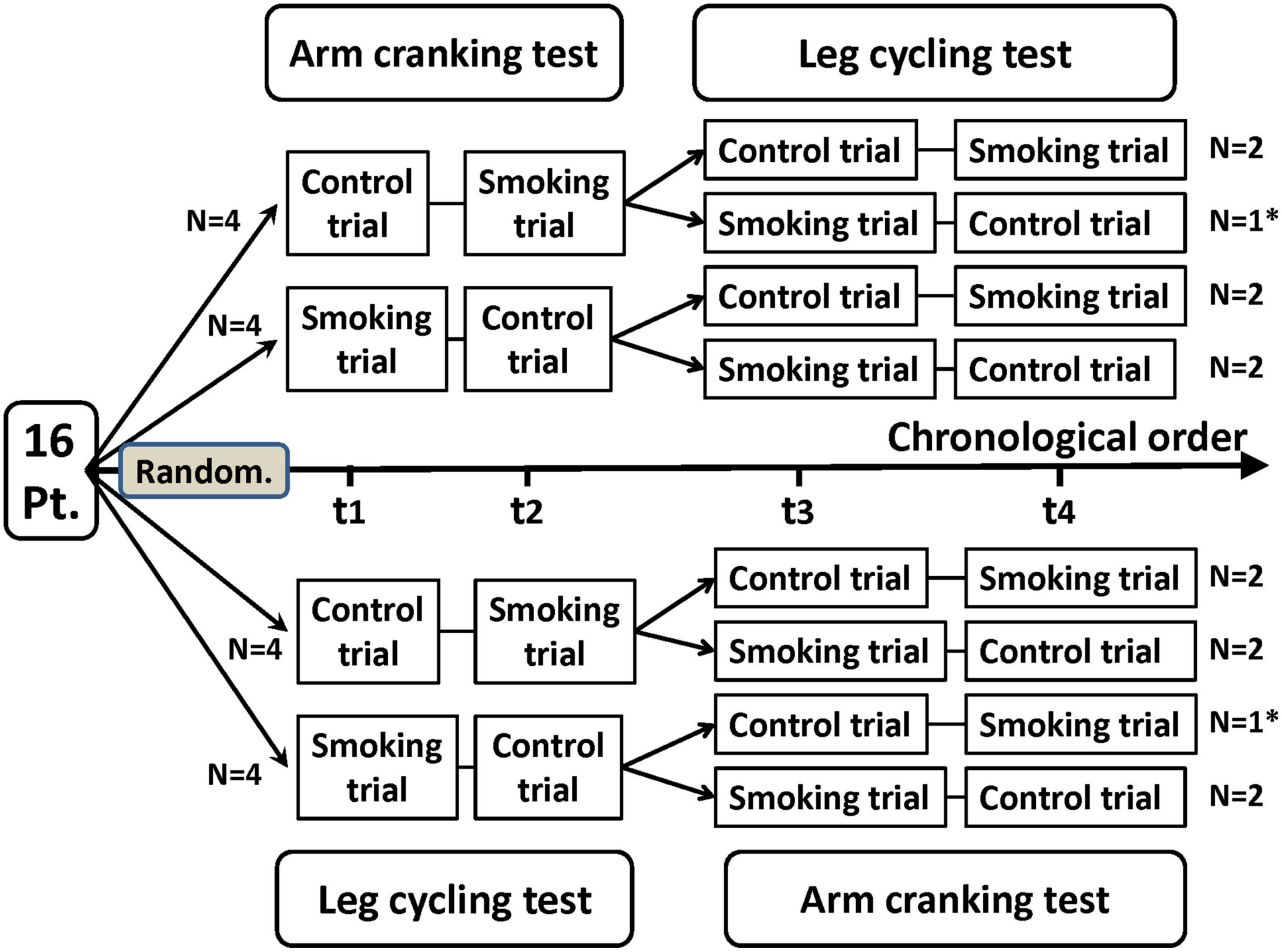
**Flowchart of a randomized crossover trial of arm cranking and leg cycling tests in 14 healthy participants**. Pt, participants; Random, randomization; t1, trial 1; t2, trial 2; t3, trial 3; t4, trial 4. ^*^Two participants discontinued the intervention because of personal reasons unrelated to the trial. These participants were not included in the analysis.

This study adopted a randomized crossover design (Figure 1). Each participant underwent 4 separate test sessions held 3–10 days apart. For two of the four test sessions, the participants were assigned to smoke two cigarettes of a popular brand (with 10 mg of tar and 0.7 mg of nicotine in each cigarette) immediately prior to each arm and leg exercise test. For the other two test sessions, they refrained from smoking for at least 8 h before exercising (i.e., the control condition). The testing sequence of the control and experimental sessions was randomly selected. The participants were instructed to avoid eating for 1 h, avoid caffeine for 4 h, and avoid alcoholic beverages for 8 h before the exercise testing; they were also instructed not to engage in strenuous exercise in the 24 h before testing. All of the test sessions were conducted in an air-conditioned laboratory with an ambient temperature between 20 and 24°C and a relative humidity of 50–60%.

### Pulmonary function tests

Pulmonary function was measured using an electronic spirometer (Vmax 29c; Sensor Medics, Yorba Linda, CA, USA) with the participant seated. Maximal inspiratory and expiratory maneuvers were performed. Forced vital capacity (FVC), forced expiratory volume in the first second (FEV_1_), forced expiratory flow in 25-75% FVC (FEF_25−75%_), peak expiratory flow (PEF), and maximum voluntary ventilation (MVV) were determined from the best of three trials before and after the participants smoked.

### Measurement and analysis of heart rate variability

**Figure 2:**
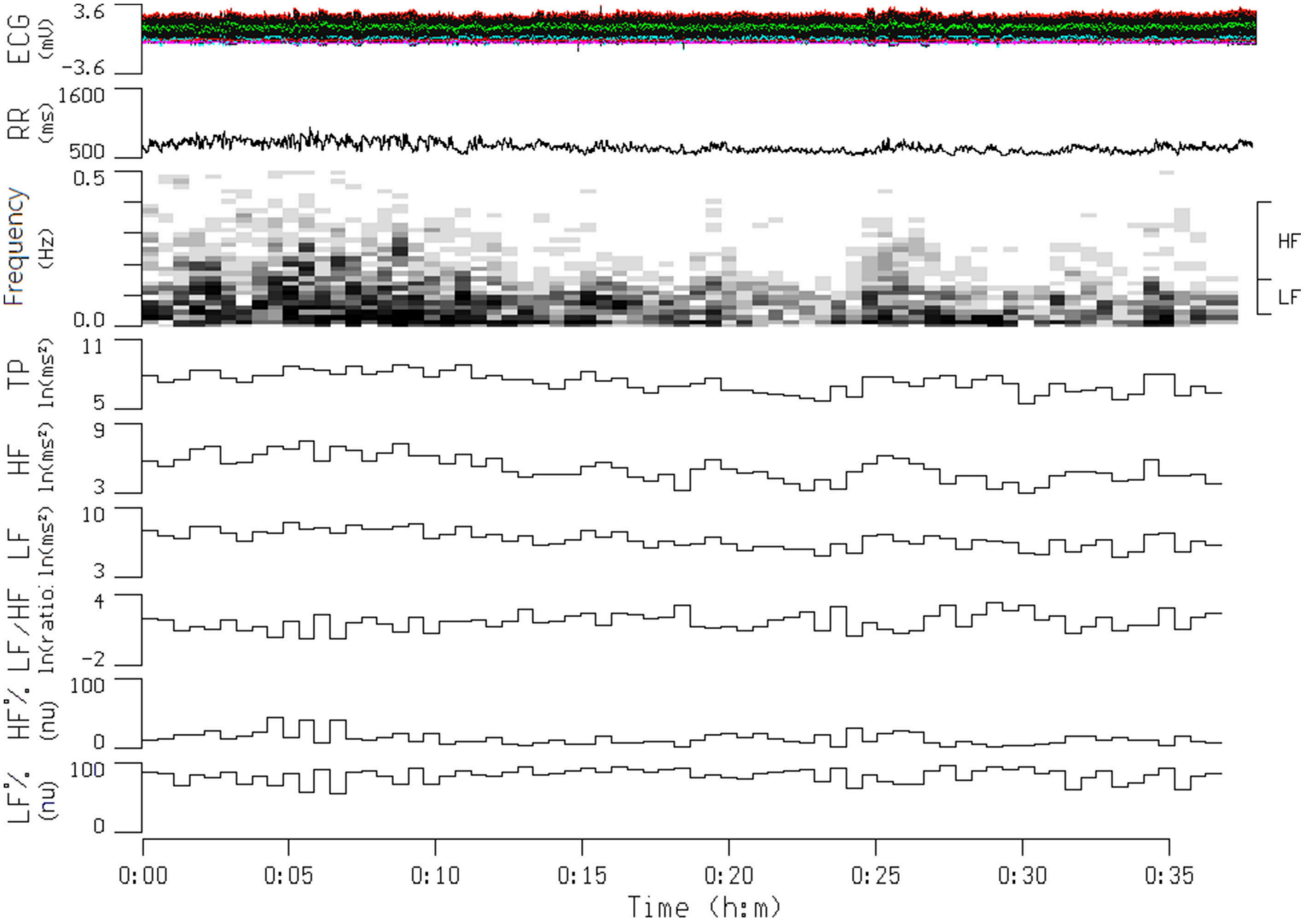
**Continuous tracing of electrocardiogram (ECG), R-R interval (RR), and power spectrogram of a participant during a transition from a before smoking period to an after smoking period**. The frequency ranges for the high-frequency (HF) and low-frequency (LF) components of RR signals are denoted on the right side of the spectrogram. Also shown are temporal displays of integrated values for the power density values of individual spectral components. This participant started to smoke at the 16-min time point, and the smoking process was finished at the 25-min time point. *TP*, total power; *LF/HF*, The ratio of low to high frequencies; *HF%*, HF in normalized unit; *LF%*, LF in normalized unit.

Heart rate variability (HRV) is the physiological phenomenon of variation in the time interval between heartbeats. It is measured by the variation in the R–R intervals [the intervals between R waves on the electrocardiogram (ECG)]. Electrophysiological signals were recorded for each participant by using a miniature physiological signal recorder (TD1; Taiwan Telemedicine Device Company, Kaohsiung, Taiwan). To compare the effects of smoking on HRV, the HRV of each participant was measured for a 10-min period beginning 10 min before the participant smoked the two cigarettes and then for another 10-min period beginning immediately after the participant smoked the two cigarettes. The length of these periods was based on the recommendations for HRV measures derived from short-term (≤ 10 min) R–R recordings (Task Force of the European Society of Cardiology and the North American Society of Pacing (Task Force of the European Society of Cardiology, 1996). An example of typical R–R interval series reflecting changes in HRV is presented in Figure 2. During the assessment period, participants were monitored using ECG leads to record their heart rate and R–R intervals. The raw ECG signals were recorded in real time after analog-to-digital conversion (8-bit) at a sampling rate of 250 Hz. The R–R intervals (in milliseconds) were calculated beat-to-beat by using a customized software program developed by Dr. Terry B. J. Kuo (Kuo et al., 1999) (the 2nd panel of Figure 2). The R-R values were resampled and interpolated at the rate of 7.11 Hz to accomplish the continuity in time domain. Frequency-domain analysis was performed using the non-parametric method of fast Fourier transform (FFT). All the signals to be analyzed were truncated into successive 30-s epochs with 50% overlapping. A Hamming window was applied to each time segment to attenuate the leakage effect (Kuo and Chan, 1993; Yang et al., 2002). For each time segment (576 s, 4096 data points), our algorithm estimated the power spectral density based on FFT. The resulting power spectrum was corrected for attenuation resulting from sampling and the application of the Hamming window (Kuo et al., 1999), and was displayed in gray scale (the 3rd panel of Figure 2). Each component of the spectrogram was subsequently quantified by the method of integration. For the HRV analysis, total power (0–0.4 Hz), low-frequency power (LF, 0.04–0.15 Hz), high-frequency power (HF, 0.15–0.4 Hz), the ratio of low to high frequencies (LF/HF), normalized LF (LF%), and normalized HF (HF%) were quantified. All the parameters were logarithmically transformed to correct for their skewed distributions (Kuo et al., 1999). Total power is a marker of autonomic nervous activity, HF power reflects parasympathetic nervous activity, and LF power reflects partial contributions from both sympathetic and parasympathetic nervous activity. To detect the sympathetic effect on HRV, total power was used to normalize LF or HF power (LF or HF%). The LF/HF was used as an index of the sympathovagal balance (Berger et al., 1989; Task Force of the European Society of Cardiology and the North American Society of Pacing Task Force of the European Society of Cardiology, 1996).

### Arm and leg exercise testing

Participants performed graded maximal exercise tests (GXTs) by using an ergometer (ANGIO with a reclining chair; Lode, Groningen, The Netherlands). The height of the ANGIO could be adjusted electrically over a range of 140 cm, making it suitable for both arm and leg ergometry. The testing protocol was preceded by a 3-min accommodation period at zero load. The workload was then increased every 2 min in steps of 16 W for the arms and 25 W for the legs, until exhaustion (Pate et al., 1991; Leicht et al., 2009). The pedaling rate was maintained at approximately 50 rpm for each participant. A pedal frequency meter with visual feedback was used by each participant to maintain the pedaling rate. Termination occurred when the participants could not continue because of exhaustion or when the target pedal rate could not be maintained for 10 s despite verbal encouragement. The end points of the graded exercise test should ideally progress until the participant reaches a level of maximal exertion. Therefore, all of the participants achieved at least two of the following criteria for determining maximum capacity: (1) A plateau in VO_2_ with an increased work rate; (2) a heart rate >85% of the age-predicted maximum (220—age); (3) a respiratory exchange ratio (RER) greater than 1.1 (Forman et al., 2010).

### Metabolic measurements and determination of the ventilatory threshold

Expired air was analyzed breath-by-breath by using an automated system (Vmax 29c; Sensor Medics, Yorba Linda, CA, USA). The exercise cardiorespiratory parameters were heart rate, VO_2_, carbon dioxide production (VCO_2_), minute ventilation (V_E_), RER, and the ventilatory equivalents for O_2_(V_E_/VO_2_) and CO_2_(V_E_/VCO_2_). V-slope and ventilatory equivalence methods were used to evaluate the ventilatory threshold (T vent) in a combined model (Forman et al., 2010). V-slope, which is the most common method, entails graphing VCO_2_ vs. VO_2_ values. The T vent is identified as the point at which there is a shift in slope along a line identified between these gas measurements. In addition, the ventilatory equivalence method is defined as the intensity of activity that causes the first rise in the V_E_/VO_2_ without a concurrent rise in the V_E_/VCO_2._ If the V-slope cannot provide a reliable T vent, it is common to review the V_E_/VO_2_ and V_E_/VCO_2_ curves to determine the T vent. Two independent evaluators with experience in exercise testing determined the T vent values.

### Data analyses

The data are presented as the mean and standard deviation (mean ± SD). Statistical differences before and after the participants smoked were calculated using paired *t*-tests in the pulmonary function test and HRV analysis. In addition, differences between the smoking and control exercise tests were calculated using paired *t*-tests for the peak power, time to exhaustion, and T vent values. However, differences in VO_2_, heart rate, and ventilation variables between the smoking and control trials during the GXTs were analyzed using trial-by-time (4 × 10) repeated-measures multivariate analysis of variance (MANOVA) with trend analysis (Holbert et al., 1990). When necessary, Bonferroni corrections were used for a *post-hoc* comparison. For the exercise test periods, VO_2_, heart rate, and V_E_ values were calculated at every 10% increment of the maximal effort time. A trend analysis was used to determine whether the response patterns differed significantly for the smoking and control trials over time. This analysis provided informative insights that were not accessible through a traditional repeated-measures design. Statistical analyses were conducted using PASW Statistics Version 18.0 (SPSS Inc., Chicago, IL, USA), and the significance level was set at *P* < 0.05.

## Results

**Figure 3:**
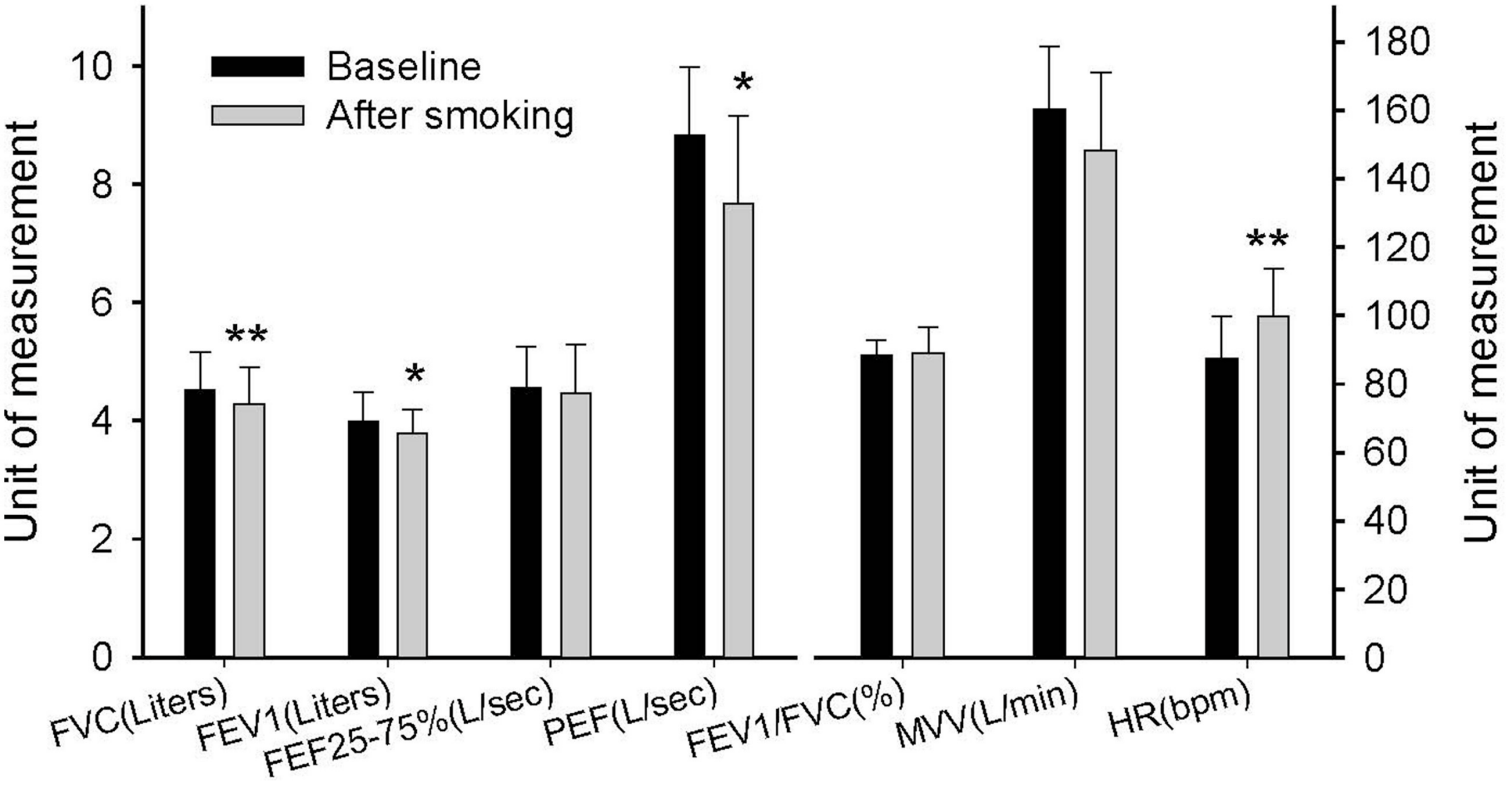
**Immediate effects of smoking on pulmonary function**. *FVC*, forced vital capacity; *FEV*_1_, forced expiratory volume in first second; *FEF*_25−75_, forced expiratory flow in 25–75% FVC; *PEF*, peak expiratory flow; *MVV*, maximum voluntary ventilation; *HR*, heart rate. Each bar is expressed as the mean ± SD, (*n* = 14). ^*^P < 0.05, ^**^P < 0.01 indicate a significant difference compared with the baseline.

Pulmonary function was first tested to serve as a baseline value in the experimental session before the GXTs. The participants were then asked to smoke two cigarettes. The second pulmonary function test was performed 5 min after the participants finished smoking in order to detect any immediate changes. Figure 3 shows that smoking caused a significant reduction in FVC (*P* = 0.003), FEV_1_ (*P* = 0.033), and PEF (*P* = 0.013). In addition, the resting heart rate increased significantly immediately after the participants smoked (*P* < 0.001). In the other experimental session, we observed immediate HRV changes after the participants smoked and before they started the GXTs. The mean R–R interval, total power, LF, and HF decreased significantly after the participants smoked (*P* < 0.05) (Table 1). However, the LF/HF ratio, HF%, and LF% did not change significantly.

**Table 1:** **Immediate effects of smoking on heart rate variability (***n*** = 14)**.

**Heart rate variability**	**Before smoking (mean ± SD)**	**After smoking (mean ± SD)**	***P*-value**
R-R (ms)	712.73±93.06	622.29±103.82	0.001
Total power [ln(ms^2^)]	7.59±0.72	6.79±1.00	0.014
LF [ln(ms^2^)]	7.03±0.79	6.27±1.08	0.026
HF [ln(ms^2^)]	5.25±0.83	4.38±1.33	0.028
LF/HF (ln ratio)	1.78±0.55	1.89±0.52	0.492
LF (%)	83.43±5.83	84.30±4.91	0.611
HF (%)	12.45±5.00	11.83±4.74	0.649

*R-R, mean of R-R intervals; Total power, marker of autonomic nervous activity; LF, low frequency power reflects both sympathetic and parasympathetic modulations; HF, high frequency reflects parasympathetic activity; LF/HF (ln ratio), the ratio of LF to HF reflects sympathovagal balance; LF%, LF in normalized unit reflects sympathetic activity; HF%, HF in normalized unit reflects sympathetic inhibition*.

Exercise performance (including peak power and time to exhaustion) was significantly higher on the LC test than on AC test in both the control and smoking trials (*P* < 0.001) (Table 2). In the LC test, the peak power and time to exhaustion were lower in the smoking trial than in the control trial (*P* = 0.005). In the AC test, although the peak power was lower in the smoking trial than in the control trial (*P* = 0.036), there was no significant difference in time to exhaustion (*P* = 0.098). In our study, smoking exerted a greater negative effect on exercise performance in the LC exercise test than in the AC exercise test.

**Table 2:** **Peak exercise test results for leg and arm ergometry according to smoking with leg-arm differences**.

**Trial type**	**Peak power (Watt)**			**Time to exhaustion (min)**		
	**Arm crank (AC)**	**Leg cycling (LC)**	**AC vs. LC *P*-value**	**Arm crank (AC)**	**Leg cycling (LC)**	**AC vs. LC *P*-value**
Control (C)	77.14 ± 9.34	158.29 ± 18.40	<0.001	8.99 ± 0.95	12.11 ± 1.42	<0.001
Smoke (S)	73.14 ± 7.88	141.36 ± 17.84	<0.001	8.51 ± 1.04	10.74 ± 1.60	<0.001
C vs. S *P*-value	0.036	0.005		0.098	0.005	

*Mean ± SD, (n = 14)*.

Table 3 shows a comparison of the T-vent performance between the smoking and control trials of the AC and LC exercise tests. For the control group, the VO_2_ values of the AC test (0.78 ± 0.26 L/min) was significantly lower than those of the LC test (1.03 ± 0.25 L/min) (*P* = 0.009). Similar results were obtained from the smoking trials: the VO_2_ values for the AC and LC tests were 0.70 ± 0.25 and 0.87 ± 0.18 L/min, respectively, (*P* = 0.029). In the AC test, no statistical difference was observed between the groups for VO_2_, V_E_, RER, and workload; however, the smoking trials displayed a trend of increased heart rate (*P* = 0.053). In the LC test, although there was no statistical difference for workload (*P* = 0.077), VO_2_ (*P* = 0.002), V_E_ (*P* < 0.001), heart rate (*P* = 0.038), and RER (*P* = 0.048) exhibited significant reductions at the T vent between the smoking and control trials. Therefore, we concluded that smoking significantly reduces the cardiorespiratory responses of the T vent in the LC exercise, whereas there were no obvious effects on the AC exercise.

**Table 3:** **Cardiorespiratory responses during leg and arm ergometry at the ventilatory threshold**.

	**Arm cranking**			**Leg cycling**		
	**Control**	**Smoking**	***P*-value**	**Control**	**Smoking**	***P*-value**
VO_2_ (L/min)	0.78±0.26	0.70±0.25	0.189	1.03±0.25	0.87±0.18	0.002
V_E_ (L/min)	22.30±6.43	21.81±8.19	0.818	26.03±3.90	21.40±2.94	<0.001
Heart rate (bpm)	116.1±17.4	127.6±20.5	0.053	118.4±11.1	111.9±9.3	0.038
RER	1.01±0.12	1.01±0.11	1.000	0.95±0.09	0.88±0.10	0.048
Workload (Watt)	41.5±13.1	42.0±13.4	0.793	69.0±19.5	58.4±12.2	0.077

*Mean ± SD, (n = 14)*.

*VO2, oxygen consumption; V_E_, ventilation; RER, respiratory exchange ratio*.

**Figure 4:**
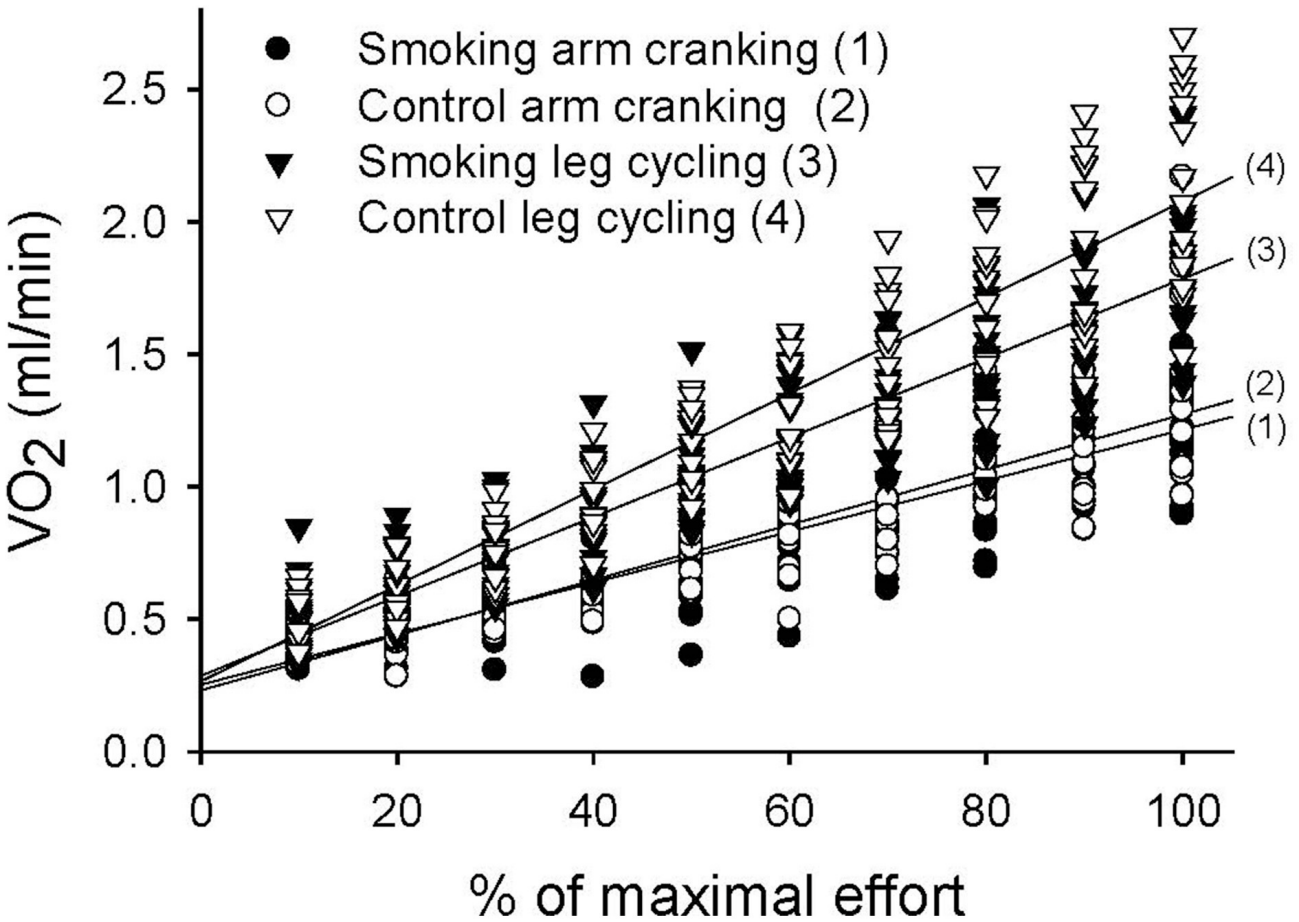
**Linear time trend for the multiple scatter plot of oxygen consumption (VO_2_) during the graded exercise tests for each trial**.

The MANOVA comparison of the VO_2_ data collected during the exercise tests resulted in a significant trial-by-time interaction [*F*_(27, 351)_ = 21.62, *P* < 0.001]; the intertrial difference in VO_2_ was also significant [*F*_(3, 39)_ = 58.46, *P* < 0.001] (Figure 4). However, the *post-hoc* analysis revealed significant differences between the control and smoking LC trials (regression line 4 vs. line 3 in Figure 4, mean difference = 0.15 ± 0.04, *P* = 0.027), between the control AC and LC trials (line 2 vs. line 4, mean difference = −0.46±0.04, *P* < 0.001), and between the smoking AC and LC trials (line 1 vs. line 3, mean difference = −0.33±0.03, *P* < 0.001). However, no significant difference was determined between the control and smoking AC trials (line 2 vs. line 1, mean difference = 0.02 ± 0.03, *P* = 1.00). A trend analysis revealed that, although all of the trials showed a linear increase in VO_2_ over time [*F*_(9, 117)_ = 317.94, *P* < 0.001], the smoking LC trial showed a more obvious decrease in its upward slope than did the control LC trial.

**Figure 5:**
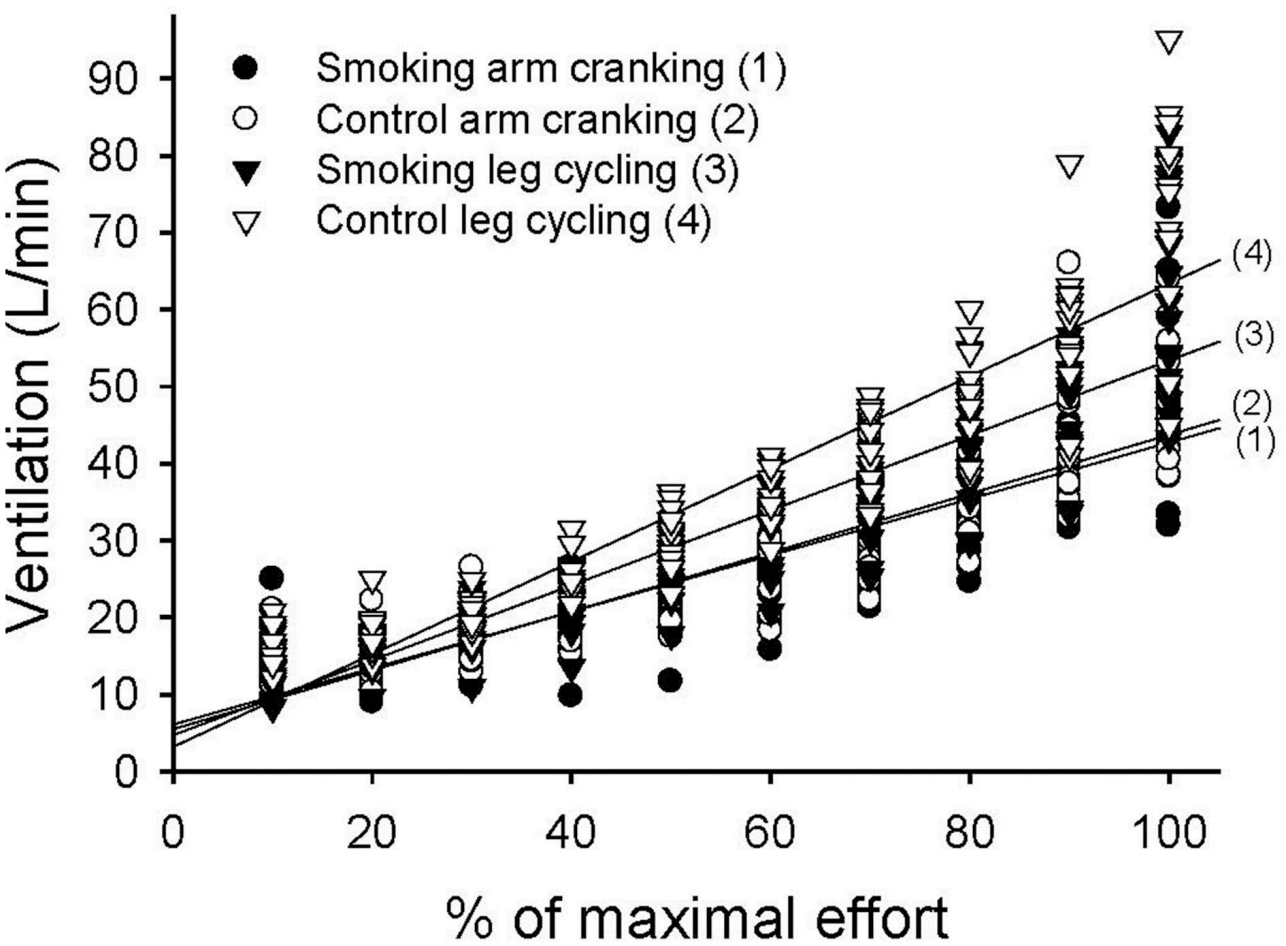
**Linear time trend for the multiple scatter plot of minute ventilation (V_E_) during the graded exercise tests for each trial**.

The variation in ventilation measured over time was significant in each trial and showed a significant trial-by-time interaction (*F* = 12.05, *P* < 0.001). The intertrial difference in ventilation was also significant (*F* = 22.61, *P* < 0.001) (Figure 5). The *post-hoc* analysis revealed significant differences between the control and smoking LC trials (regression line 4 vs. line 3 in Figure 5, mean difference = 4.82±1.49, *P* = 0.039) and between the control AC and LC trials (line 2 vs. line 4, mean difference = −9.78±0.88, *P* < 0.001); however, a significant difference was found neither between the control and smoking AC trials (line 2 vs. line 1, mean difference = 0.28 ± 0.96, *P* = 1.00) nor between the smoking AC and LC trials (line 1 vs. line 3, mean difference = −5.24±1.97, *P* = 0.118). A trend analysis revealed that, although all of the trials showed a linear increase in ventilation over time (*F* = 324.15, *P* < 0.001), the smoking LC trial showed a more obvious decrease in its upward slope compared with the control LC trial.

**Figure 6:**
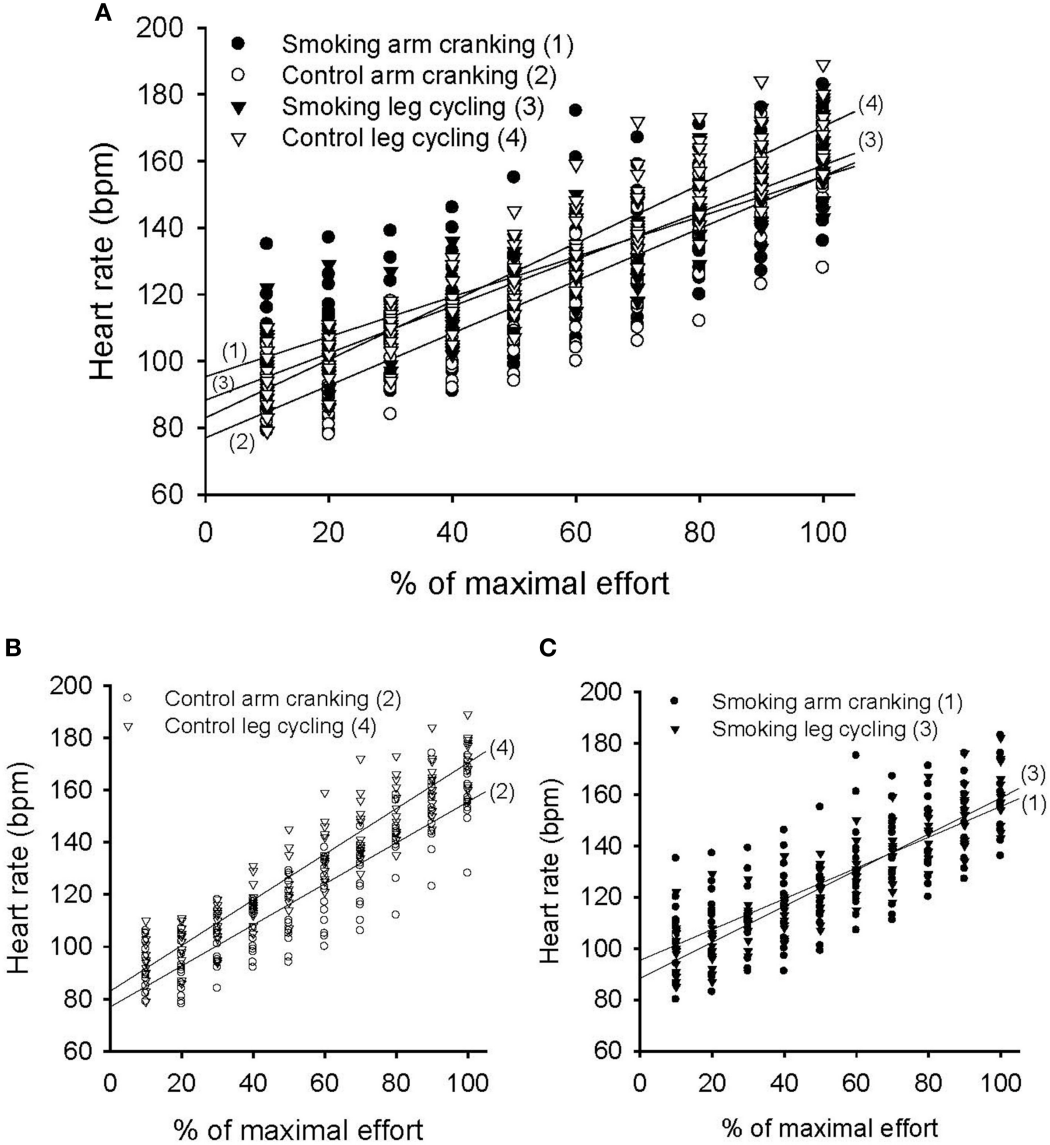
**Pattern of heart rate response during the graded exercise test in the arm cranking (AC) and leg cycling (LC) trials**. **(A)** Linear time trend for the multiple scatter plot of heart rate for each trial. **(B)** The difference of linear trend for the scatter plot between the control AC and LC trials (line 2 vs. line 4, *P* = 0.019). **(C)** The difference of linear trend between the smoking AC and LC trials (line 1 vs. line 3, *P* = 1.00).

The variation in heart rate measured over time was significant in each trial and showed a significant trial-by-time interaction (*F* = 7.44, *P* < 0.001). The intertrial difference in heart rate responses was also significant (*F* = 5.08, *P* = 0.005) (Figure 6A). The *post-hoc* analysis revealed significant differences between the control and smoking AC trials (regression line 2 vs. line 1, mean difference = −8.19±2.20, *P* = 0.015) and between the control AC and LC trials (line 2 vs. line 4 in Figure 6B, mean difference = −10.9±3.01, *P* = 0.019); however, a significant difference was found neither between the control and smoking LC trials (line 4 vs. line 3, mean difference = 4.02 ± 2.44, *P* = 0.737) nor between the smoking AC and LC trials (line 1 vs. line 3 in Figure 6C, mean difference = 1.31 ± 2.99, *P* = 1.00). A trend analysis revealed that, although all of the trials showed a linear increase in heart rate over time (*F* = 493.31, *P* < 0.001), the control LC trial showed a more pronounced upward slope compared with the control AC trial (Figure 6B). However, the trend of the control LC trial also showed a decrease in its upward slope after the participants smoked, thereby eliminating any difference between the AC and LC tests in the smoking trials (Figure 6C).

## Discussion

The present study determined that smoking two cigarettes could immediately cause a reduction in pulmonary function and an elevated resting heart rate. It also reduced parasympathetic regulation and the total power of HRV. These physiological changes had varying degrees of influence on exercise performance in the AC and LC tests. The reduction in pulmonary function (e.g., FEV_1_ and PEF) was probably due to the bronchoconstriction caused by smoking. Previous studies have indicated that increased airway resistance can last for 20–30 min (Sterling, 1967). We also determined that the immediate increase in heart rate at rest was a result of smoking's inhibition of activity in the parasympathetic nervous system (a decrease in the HF of HRV). This result is consistent with Hayano et al. (1990) regarding the short-term effects of cigarette smoking on HRV. Nevertheless, Mendonca et al. (2011) showed that smoking resulted in a decrease in vagal activity, which was paired with a shift in sympathovagal balance toward greater sympathetic dominance (both the LF/HF ratio and LF% were elevated); however, in the present study, we observed no significant change in the LF/HF ratio, LF%, or HF%. We suggest that using cigarettes with different nicotine content might have contributed to the inconsistent results. However, the Task Force of the European Society of Cardiology and the North American Society of Pacing (Task Force of the European Society of Cardiology, 1996) have shown that, in some conditions associated with sympathetic excitation, a decrease in the absolute power of the LF component is typically observed, and the resulting tachycardia is usually accompanied by a marked reduction in total power, which is comparable to the findings derived from the data in our study. Moreover, the reduction in total power influenced by LF and HF power occurred in the same direction and prevented the appreciation of fractional energy distribution, which would explain why the heart rate increase during exercise was flatter than expected in the present study. This result could be caused by the total power significantly decreasing after the participants smoked, which would be consistent with Mendonca et al. (2011).

In the control trials of the GXTs, the peak power, time to exhaustion, and maximal responses of the heart rate, VO_2_, and V_E_ were lower for the AC exercise test than for the LC test. This result is consistent with previous studies (Pate et al., 1991; Tulppo et al., 1999; Yasuda et al., 2002; Helge, 2010; Orr et al., 2013) because the arm muscle mass involved in AC activity is small and muscular fatigue generally occurs before the cardiorespiratory system reaches its peak (Kang et al., 2004). The plateauing of VO_2_ was generally unapparent in the AC tests. For the experimental trials during the GXTs, the phenomenon of higher exercise performance during the LC exercise compared with the AC exercise does not change with smoking. However, differences in cardiorespiratory responses between the LC and the AC exercises became smaller and even disappeared after the participants smoked. For example, no statistical difference was observed in V_E_ and heart rate between theAC and the LC exercise tests after the participants smoked (line 1 vs. line 3 in Figures 5, 6C). Because the lower-body musculature has a greater percentage of slow-twitch fibers compared with the upper-body musculature (Johnson et al., 1973), we suggest that slow-twitch fibers were more sensitive to the negative effects of smoking. In addition, in the LC exercise tests, time to exhaustion and the slopes of the VO_2_ and V_E_ values decreased significantly after the participants smoked. Our results are consistent with previous studies (Klausen et al., 1983; Hirsch et al., 1985; Rotstein and Sagiv, 1986). However, the unique finding of the present study is that there was no significant change between the smoking and control trials of the AC exercise tests (Figures 4, 5). Therefore, we conclude that smoking-induced reductions in exercise performance occur only when cardiopulmonary systems reach their maximal capacities. In the AC exercise tests, the upper-body musculature was exhausted before the cardiorespiratory systems reached their capacities.

Johnson et al. (1973) reported that the upper-body musculature has a higher proportion of Type II fibers compared with the lower-body musculature. According to Ahlborg and Jensen-Urstad (1991), AC exercise induces a greater increase in muscle glycogen use than does LC exercise at the same percentage of VO_2*peak*_, thus resulting in a higher net lactate release from exercising muscles. Although muscle glycogen and lactate concentrations were not measured in the present study, one explanation for higher carbohydrate oxidation during AC exercise might be that the aerobic capacity of the skeletal muscles tends to be lower in the arms because they are used less than the legs in daily activities. In this study, we also determined the T vent between the AC and LC exercise tests and the effect of smoking on the T vent. Our results show that the T vent of the control AC exercise test was lower than that of the LC exercise test. In addition, smoking significantly reduced the T vent of the LC exercise test but not that of the AC exercise test. These results indirectly support the notion that the aerobic capacity of arm skeletal muscles tends to be lower than that of the leg muscles because of the higher proportion of Type II muscle fibers in arm muscles. Consequently, the effect of smoking on the T vent is more pronounced in LC than in AC exercise tests.

Previous studies have revealed that arm exercise interferes with the respiratory muscles. Using the arms might interfere with inspiration by limiting the ability of the diaphragm to descend or of the rib cage to expand (Cerny and Ucer, 2004). The maximal ventilatory capacity of AC exercises is fundamentally limited by this factor, which contributes to a lower ventilatory capacity in the arm musculature than in the leg musculature and deteriorates exercise performance in extreme AC exercises. Consequently, the arm musculature commonly fatigues before reaching maximal cardiopulmonary function during GXTs, causing exercise performance to be significantly lower on the AC tests than on the LC tests. In addition, the negative effect of smoking on the maximal aerobic exercise test of the AC trials may have been diluted.

This study had certain limitations. It investigated the immediate responses of smoking on moderately trained people during maximal AC and LC exercise tests. The interference caused by AC exercise on the respiratory muscles differs little between moderately trained people and elite athletes; however, elite athletes certainly have a higher aerobic capacity. Therefore, we cannot determine what impact smoking has on the AC aerobic performance of elite athletes.

## Conclusions and practical implications

This study verifies that smoking can significantly decrease LC exercise performance by reducing heart rate reserves (i.e., increased resting heart rate and decreased heart rate during maximal exercise) and HRV activity; these factors can all affect maximal VO_2_ during exercise. In addition, smoking immediately reduces pulmonary function by decreasing FVC, FEV_1_, and PEF. Consequently, maximal V_E_ during LC exercise is reduced. These negative effects of smoking caused significant performance reductions in LC exercise tests. However, the effects of smoking on AC exercises were not as pronounced.

Many people, including occupational workers, physically challenged people, and athletes, engage in work activities that predominantly entail using the upper or lower extremities. For example, canoeing and wheelchair sports predominantly use the arms, whereas bicycle racing predominantly uses the legs. Consequently, comparing differences in the effect of smoking on arm and leg exercises can both qualitatively and quantitatively clarify variations in exercise capacity, the conditions for specific activities, and sport performance.

### Conflict of interest statement

The authors declare that the research was conducted in the absence of any commercial or financial relationships that could be construed as a potential conflict of interest.

## References

[B1] Ahlborg G. Jensen-Urstad M. (1991). Metabolism in exercising arm vs. leg muscle. Clin. Physiol. 11, 459–468. 10.1111/j.1475-097X.1991.tb00818.x 1934942

[B2] Berger R. D. Saul J. P. Cohen R. J. (1989). Transfer function analysis of autonomic regulation. I. Canine atrial rate response. Am. J. Physiol. 256, H142–H152. 2912176 10.1152/ajpheart.1989.256.1.H142

[B3] Cerny F. J. Ucer C. (2004). Arm work interferes with normal ventilation. Appl. Ergon. 35, 411–415. 10.1016/j.apergo.2004.05.001 15246879

[B4] Forman D. E. Myers J. Lavie C. J. Guazzi M. Celli B. Arena R. (2010). Cardiopulmonary exercise testing: relevant but underused. Postgrad. Med. 122, 68–86. 10.3810/pgm.2010.11.2225 21084784 PMC9445315

[B5] Hawari F. I. Obeidat N. A. Ayub H. Ghonimat I. Eissenberg T. Dawahrah S.. (2013). The acute effects of waterpipe smoking on lung function and exercise capacity in a pilot study of healthy participants. Inhal. Toxicol. 25, 492–497. 10.3109/08958378.2013.806613 23905967

[B6] Hayano J. Yamada M. Sakakibara Y. Fujinami T. Yokoyama K. Watanabe Y.. (1990). Short- and long-term effects of cigarette smoking on heart rate variability. Am. J. Cardiol. 65, 84–88. 10.1016/0002-9149(90)90030-5 2294686

[B7] Helge J. W. (2010). Arm and leg substrate utilization and muscle adaptation after prolonged low-intensity training. Acta Physiol. 199, 519–528. 10.1111/j.1748-1716.2010.02123.x 20345410

[B8] Hirsch G. L. Sue D. Y. Wasserman K. Robinson T. E. Hansen J. E. (1985). Immediate effects of cigarette smoking on cardiorespiratory responses to exercise. J. Appl. Physiol. 58, 1975–1981. 4008417 10.1152/jappl.1985.58.6.1975

[B9] Holbert D. Chenier T. C. O'Brien K. F. (1990). Trend analysis for repeated measures designs. Med. Sci. Sports Exerc. 22, 871–878. 10.1249/00005768-199012000-00022 2132745

[B10] Iyawe V. I. Ejindu C. N. Ebomoyi M. I. Oboh H. A. (2007). The effect of a single cigarette puff on air flow in the lungs. J. Med. Biomed. Res. 6, 4–12. 10.4314/jmbr.v6i1.10698

[B11] Johnson M. A. Polgar J. Weightman D. Appleton D. (1973). Data on the distribution of fiber types in thirty-six human muscles. An autopsy study. J. Neurol. Sci. 18, 111–129. 10.1016/0022-510X(73)90023-3 4120482

[B12] Kang J. Hoffman J. R. Wendell M. Walker H. Hebert M. (2004). Effect of contraction frequency on energy expenditure and substrate utilization during upper and lower body exercise. Br. J. Sports Med. 38, 31–35. 10.1136/bjsm.2002.002121 14751942 PMC1724755

[B13] Klausen K. Andersen C. Nandrup S. (1983). Acute effects of cigarette smoking and inhalation of carbon monoxide during maximal exercise. Eur. J. Appl. Physiol. 51, 371–379. 10.1007/BF00429074 6685036

[B14] Kuo T. B. J. Chan S. H. H. (1993). Continuous, on-line, real-time spectral analysis of systemic arterial pressure signals. Am. J. Physiol. 264, H2208–H2213. 8322952 10.1152/ajpheart.1993.264.6.H2208

[B15] Kuo T. B. J. Lin T. Yang C. C. H. Li C. L. Chen C. F. Chou P. (1999). Effect of aging on gender differences in neural control of heart rate. Am. J. Physiol. 277, H2233–H2239. 10600841 10.1152/ajpheart.1999.277.6.H2233

[B16] Leicht A. S. Sealey R. M. Sinclair W. H. (2009). The reliability of VO_2peak_ determination in healthy females during an incremental arm ergometry test. Int. J. Sports Med. 30, 509–515. 10.1055/s-0029-1202351 19455479

[B17] Mendonca G. V. Pereira F. D. Fernhall B. (2011). Effects of cigarette smoking on cardiac autonomic function during dynamic exercise. J. Sports Sci. 29, 879–886. 10.1080/02640414.2011.572991 21547834

[B18] Orr J. L. Williamson P. Anderson W. Ross R. McCafferty S. Fettes P. (2013). Cardiopulmonary exercise test: arm crank vs cycle ergometry. Anaesthesia 68, 497–501. 10.1111/anae.12195 23573845

[B19] Pate R. R. Blair S. N. Durstine J. L. Eddy D. O. Hanson P. Painter P. (1991). Guidelines for exercise test administration, in ACSM Guidelines for Exercise Testing and Prescription, ed American College of Sports Medicine (Philadelphia, PA: Lea & Febiger), 55–91.

[B20] Power S. K. Howley E. T. (eds.). (2004). Circulatory adaptations to exercise, in Exercise Physiology: Theory and Application to Fitness and Performance (New York, NY: McGraw-Hill), 164–191.

[B21] Rotstein A. Sagiv M. (1986). Acute effect of cigarette smoking on physiologic response to graded exercise. Int. J. Sports Med. 7, 322–324. 10.1055/s-2008-1025784 3804539

[B22] Seppänen A. (1977). Physical work capacity in relations to carbon monoxide inhalation and tobacco smoking. Ann. Clin. Res. 9, 269–274. 616212

[B23] Sobol B. J. Van Voorhies L. Emirgil C. (1977). Detection of acute effects of cigarette smoking on airway dynamics: A critical and comparative study of pulmonary function tests. Thorax 32, 312–316. 10.1136/thx.32.3.312 882945 PMC470607

[B24] Sterling G. M. (1967). Mechanism of bronchoconstriction caused by cigarette smoking. Br. Med. J. 3, 275–277. 10.1136/bmj.3.5560.275 6028730 PMC1842135

[B25] Task Force of the European Society of Cardiology the North American Society of Pacing and Electrophysiology. (1996). Heart rate variability: standards of measurement, physiological interpretation and clinical use. Circulation 93, 1043–1065. 10.1161/01.CIR.93.5.1043 8598068

[B26] Tulppo M. P. Makikallio T. H. Laukkanen R. T. Huikuri H. V. (1999). Differences in autonomic modulation of heart rate during arm and leg exercise. Clin. Physiol. 19, 294–299. 10.1046/j.1365-2281.1999.00180.x 10451789

[B27] Yang C. C. H. Lai C. W. Lai H. Y. Kuo T. B. J. (2002). Relationship between electroencephalogram slow-wave magnitude and heart rate variability during sleep in humans. Neurosci. Lett. 329, 213–216. 10.1016/S0304-3940(02)00661-4 12165415

[B28] Yasuda N. Ruby B. C. Gaskill S. E. (2002). Substrate utilization during arm and leg exercise relative to the ventilatory threshold in men. J. Sports Med. Phys. Fitness 42, 403–408. Available online at: http://www.minervamedica.it/en/journals/sports-med-physical-fitness/article.php?cod=R40Y2002N04A0403 12391433

